# Study of Water- and Organic-Soluble Extracts from *Trichosanthes* on Type 1 Diabetes Mellitus

**DOI:** 10.1155/2022/3250016

**Published:** 2022-02-17

**Authors:** Bo Zhang, Yanli Yuan, Jie Xin, Min Chen, Zhen Wang, Xinpeng Li, Tao Xue

**Affiliations:** ^1^College of Pharmacy, Linyi University, Linyi, Shandong, China; ^2^Chinese Academy of Traditional Chinese Medicine, China

## Abstract

This study investigates the effects of the water-soluble and organic-soluble *Trichosanthes* extracts on the hyperglycemic condition in streptozotocin- (STZ-) induced diabetic rats. The blood glucose levels, body weights, water intake, and urine volumes of rats in different experimental groups were monitored throughout the experiment, and the results obtained indicate that the two extracts can effectively reduce blood sugar levels, increase body weights, and improve water intake and urine volumes in diabetic rats. Based on blood biochemical analyses, the two extracts play an important role in regulating the diabetes-induced lipid metabolism disorder, increasing the levels of insulin and C-peptide, and alleviating the symptoms of diabetes. The variation in the liver glycogen contents of the water-soluble fraction and ethanol fraction groups suggests that the mechanisms underlying the hypoglycemic effects of the two extracts are different. Indeed, the water-soluble fraction alleviates diabetes symptoms in rats mainly by antioxidative activity, unlike the ethanol fraction.

## 1. Introduction

Type 1 diabetes mellitus (T1DM) is an organ-specific disease mediated by autoimmune deficiency and the selective destruction of islet B cells [1, 2]. T1DM accounts for about 10% of diabetes mellitus [3], and its incidence rate has increased by about 4% per year in the last few years, especially in children and adolescents [4]. Despite the optimization of insulin administration, the disease leads to chronic hyperglycemia and hyperglycemic episodes in most patients [5, 6]. These conditions are associated with severe clinical symptoms and complications such as ketoacidosis and hypoglycemic coma, and they shorten the life expectancy of patients by more than 10 years. Unfortunately, the oral hypoglycemic drugs used in clinical treatment cannot effectively control T1DM, and only continuing insulin injections can be used to balance blood glucose levels in patients. However, there is hope that the in-depth study of T1DM pathogenesis may enable the development of an immune intervention that can prevent or reverse the disease [7, 8]. During the past 30 years, many clinical trials of immune intervention have been carried out, and several treatments have been tested, including immunomodulatory drugs, regulatory T cells, stem cell therapy, and islet transplantation [9, 10]. Although these trials can delay the decline of islet B cell function or limit the duration of insulin therapy by inhibiting autoimmune response or islet B cell replacement, they still have their own limitations [11–13]. In effect, very few efficient drugs are currently available for the treatment of T1DM [14].

In ancient times, diabetes was called Xiaoke lesion, and it was treated using different kinds of traditional Chinese medicines. The Shennong Materia Medica reported that among the used medicines, *Trichosanthes* (the root of *Trichosanthes kirilowii Maxim*. in Cucurbitaceae family) can relieve thirst, body heat, distemper, great heat, tonify deficiency and ease the middle, and continue to eliminate injury. Moreover, in Li's Compendium of Materia Medica, *Trichosanthes* was record as the holy medicine for relieving thirst [15]. *Trichosanthes* is actually included in 143 out of 592 traditional Chinese medicine prescriptions used for the treatment of diabetes, and thus, it is the fourth-ranking active ingredient. In addition, the use frequency of *Trichosanthes*-containing prescriptions exceeded 50%, which shows that in traditional Chinese medicine, this plant is of great significance for the treatment of diabetes [16]. Consequently, it can potentially be used to develop effective diabetes drugs and therapies.

In this study, we assess the immune intervention effects of the water and alcohol extracts of *Trichosanthes* in an STZ-induced type 1 diabetic rat model. Through this study, we could develop more therapeutic drugs for T1DM. More importantly, our research data is a supplement to the previous research, which can provide more accurate research data for later clinical development and application.

## 2. Materials and Methods

### 2.1. Materials

Male Sprague-Dawley (SD) rats (6 weeks old, 180~200 g) were provided by the Shandong University Experimental Animal Center (Shandong, China). STZ was purchased from Sigma-Aldrich (S0130-1G; St. Louis, MO, USA). All of the other chemicals and reagents were obtained from general commercial sources (Solarbio, Beijing, China), and they were used as received, unless otherwise specified. *T. kirilowii Maxim*. was cultured in Linyi (Shandong Province, China) and harvested in August 2019. The drug was authenticated by a botanist at the Shandong University of traditional Chinese Medicine, Jinan. The fresh roots were washed, sundried, and stored in plastic bags at room temperature until further use. The antibodies were purchased from Affinity Inc. (Rocky Hill, NJ, USA).

### 2.2. Preparation of the Water-Soluble and Ethanol Fractions of *T. kirilowii*

*T. kirilowii Maxim*. roots were sliced, crushed, and boiled in pure water for 2 hours (the ratio of material to liquid =1 : 10), and the resulting aqueous solution was purified with diatomite and concentrated. Subsequently, the concentrate was freeze-dried to obtain the water-soluble fraction. To prepare the ethanol fraction, the *T. kirilowii* Maxim. roots were first boiled in anhydrous ethanol (*T* > 78°C, ethanol reflux, the ratio of material to liquid = 1 : 5); then, the solution was concentrated and vacuum dried.

### 2.3. Animal Experiments

The animal experiments were conducted in accordance with the Chinese National Guide for the Care and Use of Laboratory Animals and approved by the ethics committee of Linyi University. The male SD rats were kept in a pathogen-free animal room where they were subjected to 12 : 12 h light/dark cycles. The animals were randomly divided into four groups (sham, model, water-soluble fraction, and ethanol fraction groups), with 10 rats in each group, and they were acclimated to the environment for one week before experimentation. After the acclimation period, the rats were administered with daily injections of 55 mg/kg body weight STZ in normal saline [17]. The blood glucose levels were measured to confirm that the diabetes model had been successfully established (blood glucose >16.5 mmol/L), and the mortality rates corresponding to the four groups were also recorded. In addition to STZ, the rats in the water-soluble fraction group were administered with 200 mg/kg/day *T. kirilowii* water extract solution by gavage. This solution was prepared by dissolving the freeze-dried water extract in pure water and then filtering through a 0.22 *μ*m membrane, and it was stored at 4°C prior to use. Meanwhile, the ethanol fraction group was given daily doses of 20 mg/kg ethanol extract solution (extract dissolved in a 2 : 8 *v*/*v* ethanol : water mixture), also by gavage. The rats in the sham group and diabetes mellitus (model) group were administered with normal saline by gavage, according to the same schedule. After five weeks, the rats were weighed; then, they were anesthetized with phenytoin sodium. Their livers and skeletal muscles were collected, and their blood was collected from the celiac artery. The serum was obtained by centrifugation at 2250 × *g* and 4°C for 20 min, and it was used for biochemical analysis.

### 2.4. Effects of the Water-Soluble and Ethanol Fractions on OGTT

The oral glucose tolerance test (OGTT) is an important indicator of diabetes; so, it was performed on the experimental animals in this study [18]. One day before the end of the experiment, the rats in all groups fasted for 6 h; then, they were gavage-fed with 50% glucose solution at the dose of 1 mL/100 g body weight (equivalent to 5 g of glucose per 1 kg body weight). Subsequently, the blood glucose levels were measured at 0, 30, 60, 120, and 180 min after intragastric administration, and the OGTT curves were recorded and drawn.

### 2.5. Monitoring of Body Weight, Blood Glucose, Water Consumption, and Urine Volume

The body weights and blood glucose levels of rats in different groups were measured and recorded every week. Similarly, changes in the amount of water consumed were monitored, and the volumes of excreted urine were measured using a metabolic cage. The curves showing the variation of these parameters in each group were drawn.

### 2.6. Blood Biochemical Analysis

An automated chemistry analyzer (Synchron CX5, Beckman Coulter, Brea, CA) was used to assess the triglyceride (TG), total cholesterol (TC), low-density lipoprotein cholesterol (LDL-C), high-density lipoprotein cholesterol (HDL-C), pyruvate, and C-peptide levels in the plasma of rats. The serum insulin level was detected by ELISA.

### 2.7. Histologic Examination

Upon removal, the liver and skeletal muscles of each rat were immediately fixed in 4% formaldehyde. After routine paraffin embedding, the sample was cut into 5 *μ*m sections and stained with periodic acid-silver (PAS) for the detection of histopathological alterations and glycogen changes [19].

### 2.8. Detection of Oxidative Stress Activation in the Liver

The rat livers collected at the end of the experiment were centrifuged, and changes in the superoxide dismutase (SOD), malondialdehyde (MDA), glutathione (GSH), glutathione peroxidase (GSH-Px), and catalase (CAT) levels were detected in the supernatant using the appropriate kits (Solarbio, Beijing, China).

### 2.9. Antioxidant Activity of the *Trichosanthes* Extracts

The in vitro antioxidant activity of the *Trichosanthes* extracts was determined by micro method, using the appropriate kits. The removal rates of hydroxyl radicals (BC1325, Solarbio, Beijing, China), superoxide anion radicals (BC1415, Solarbio), and DPPH radicals (BC4775, Solarbio) were assessed.

### 2.10. Statistical Analysis

All of the quantitative data presented in this text were expressed as mean ± SD. A software was used (GraphPad Prism 8.0) for the statistical analysis, and variations among groups were assessed by one-way analysis of variance (ANOVA), followed by Dunnett's test. Meanwhile, nonparametric data were evaluated using the Mann–Whitney *U* test. Differences were considered to be statistically significant and very significant at ^∗^*p* <0.05 (∗/#) and ^∗^*p* <0.01 (∗∗/##), respectively.

## 3. Results

### 3.1. Effects of the Water-Soluble and Ethanol Fractions on Diabetes

**Figure 1:**
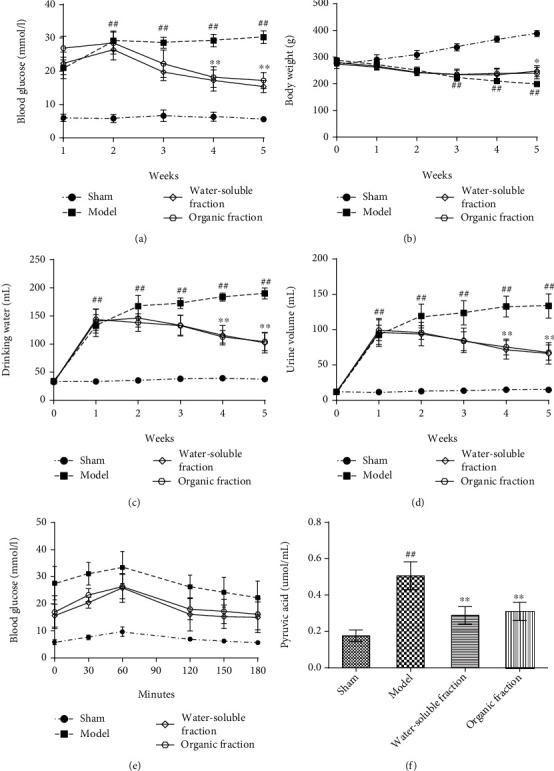
Variations in (a) blood glucose levels, (b) body weights, (c) water consumption, and (d) urinary volumes (24 h urine) of rats in different groups. (e) OGTT results and (f) pyruvic acid contents. ^##^*p* < 0.01 vs. the sham group; ^∗∗^*p* < 0.01 vs. the model group, *n* ≥ 6.

The body weights, blood glucose levels, water consumption, and urine volumes of rats were detected before the experiment, and they were found to be normal in all rats. After STZ injection, these parameters were tested once a week, and based on the results obtained, the blood glucose levels in sham rats did not significantly change, but the weights of these rats increased continuously (^∗∗^*p* < 0.01 vs. the model group). Meanwhile, the weights of rats in the model group decreased gradually (^##^^∗^*p* <0.01 vs. the sham group), their blood glucose levels remained high, and their water consumption and urine volumes increased sharply (^##^^∗^*p* <0.01 vs. the sham group; ^∗∗^*p* < 0.01 vs. the model group). These symptoms are typical of type 1 diabetes. The body weights of rats in the water-soluble and ethanol fraction groups increased at the end of the experiment, and their blood glucose decreased to levels lower than the model standard level (16.5 mmol/L) (Table 1 and Figures 1(a)–1(d)). Therefore, it might be concluded that both the water-soluble and ethanol extracts of *Trichosanthes* can reduce the blood glucose levels in diabetic rats and effectively control the development of T1DM.

### 3.2. Effects of Water-Soluble and Ethanol Fractions on OGTT and Pyruvic Acid

To verify the effects of water-soluble and ethanol *Trichosanthes* extracts on blood glucose regulation in diabetic rats, the OGTT experiment was performed. In this experiment, the blood glucose levels of rats were measured at 0, 30, 60, 120, 150, and 180 min after intragastric administration of 50% glucose solution. The results obtained showed that the sham group rats exhibited normal blood glucose levels after 180 min of glucose administration; however, the levels of the model group rats remained high. In the water-soluble fraction and ethanol fraction groups, the levels of blood glucose increased at first and then decreased to the normal level detected before the beginning of the experiment (Table 2 and Figure 1(e)).

The content of pyruvate in sham rat serum was found to be normal, but the model group rats exhibited significantly increased pyruvate contents in their serum. The *Trichosanthes* extract groups presented serum pyruvate levels similar to those of the sham group but significantly different from those of the model group (^∗∗^*p* < 0.01, Figure 1(f)).

### 3.3. Blood Biochemical Analysis

**Figure 2:**
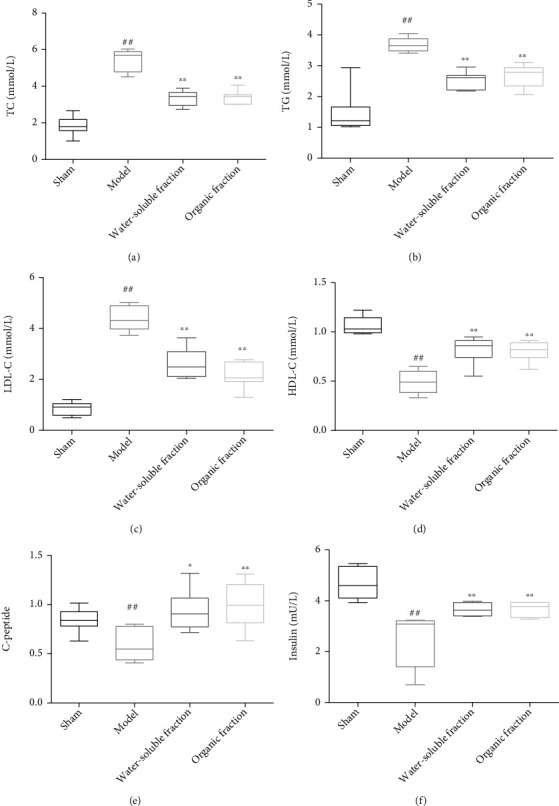
Serum biochemical analysis. Variations in (a) TC, (b) TG, (c) LDL-C, and (d) HDL-C blood levels at the end of the experiment (^##^*p* < 0.01 vs. the sham group; ^∗∗^*p* < 0.01 vs. the model group, *n* ≥ 6). (e) Variations in C-peptide serum level at the end of the experiment (^##^*p* < 0.01 vs. the sham group; ^∗^*p* < 0.05 and ^∗∗^*p* < 0.01 vs. the model group, *n* ≥ 6). (f) Variations in insulin serum level at the end of the experiment (^##^*p* < 0.01 vs. the sham group; ^∗∗^*p* < 0.01 vs. the model group, *n* ≥ 6).

Compared to the sham group, the levels of TC, TG, and LDL-C in the model group were significantly higher, while those of HDL-C were significantly lower (Figures 2(a)–2(d)). The levels of these lipids in the extract groups were similar to those in the sham group but significantly different from those of in the model group (^##^*p* <0.01 vs. the sham group), which indicated that these extracts can effectively regulate the blood lipid metabolism in diabetic rats. Using the ELISA kit, the contents of C-peptide and insulin in rat serum were also detected, and the results indicated that STZ injection significantly decreased these contents in the model group rats (Figures 2(e) and 2(f)). This showed that the insulin secretion in diabetic rats was insufficient. However, treatment with the water-soluble or ethanol extracts of *Trichosanthes* can restore the normal serum levels of C-peptide and insulin, which confirmed the positive role of these extracts in regulating insulin secretion and reducing the blood glucose concentration in diabetic rats (^∗^*p* < 0.05 and ^∗∗^*p* < 0.01 vs. the model group).

### 3.4. Histologic Examination

**Figure 3:**
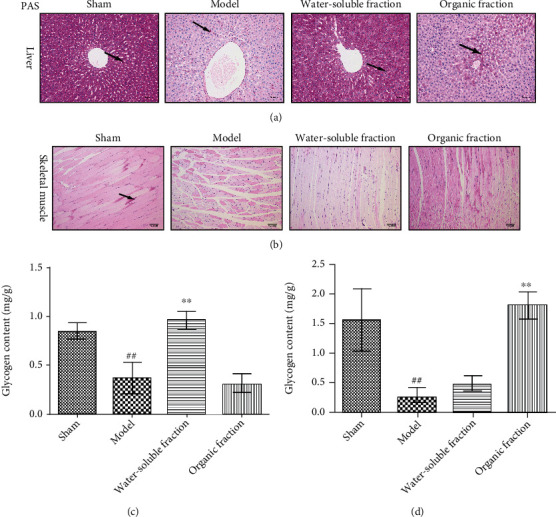
PAS staining to observe glycogen deposition in the (a) livers and (b) skeletal muscles of rats. (c, d) Data statistics of glycogen content (^##^*p* < 0.01 vs. the sham group; ^∗∗^*p* < 0.01 vs. the model group, *n* ≥ 6).

The contents of glycogen in the livers and skeletal muscles of rats were observed by PAS staining (Figures 3(a) and 3(b)). The microscopic images showed that the liver glycogen in the model group was significantly reduced compared to that in the sham group and that fatty degeneration occurred. The administration of the water-soluble *Trichosanthes* fraction increased the content of glycogen in the liver, but the ethanol fraction had no significant effect on this content. Moreover, both *Trichosanthes* extracts alleviated the skeletal muscle atrophy observed in diabetic rats (model group). However, only the ethanol extract increased the content of glycogen in rat skeletal muscles, compared to the model group (^∗∗^*p* < 0.01 vs. the model group). It should be noted that the liver and skeletal muscle glycogen contents detected using PAS staining were similar to those measured using the kit (Figures 3(c) and 3(d)).

### 3.5. *In Vivo* and *In Vitro* Antioxidant Activity

**Figure 4:**
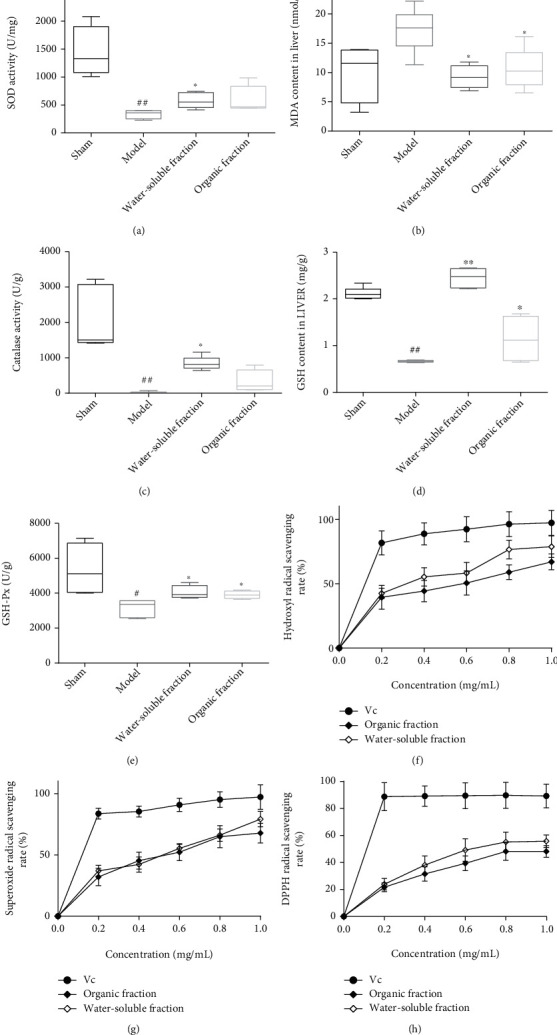
Variations in (a) SOD activity, (b) liver MDA level, (c) catalase activity, (d) liver GSH level, and (e) liver GSH-Px level at the end of the experiment (^#^*p* < 0.05 and ^##^*p* < 0.01 vs. the sham group; ^∗^*p* < 0.05 and ^∗∗^*p* < 0.01 vs. the model group, *n* ≥ 6). (f) Hydroxyl radical, (g) superoxide free radical, and (h) DPPH radical scavenging rates (Vc: ascorbic acid).

At the end of the experiment, the antioxidant activity of *Trichosanthes* in rat livers was tested *in vivo*. Based on the SOD activity test, the livers of the model group rats exhibited significantly lower contents of SOD than the livers of the sham group rats (^##^*p* <0.01 vs. the sham group). Although the administration of the water-soluble fraction inhibited the diabetes-induced decrease in SOD (^∗^*p* < 0.05 vs. the model group), treatment with the ethanol fraction had no appreciable effect on the SOD content in rat livers (Figure 4(a)). Moreover, the MDA level detected in the model group was significantly higher than that measured in the sham group (^#^*p* <0.05 vs. the sham group), and both *Trichosanthes* extracts decreased this level (Figure 4(b); ^∗^*p* < 0.05 vs. the model group). The liver catalase content in the sham group was very high, but it was significantly reduced in the model group. Although the water-soluble fraction countered the effect of diabetes on the catalase content, the ethanol fraction did not appreciably change it (Figure 4(c); ^##^*p* <0.01 vs. the sham group; ^∗^*p* < 0.05 vs. the model group). Finally, the GSH and GSH-Px levels in the livers of the model group rats were significantly less than those detected in the sham group rats (Figures 4(d) and 4(e); ^#^*p* <0.05 and ^##^*p* <0.01 vs. the sham group; ^∗^*p* < 0.05 and ^∗∗^*p* < 0.01 vs. the model group), but the administration of the water-soluble or ethanol fractions obviously increased these levels.

The *in vitro* antioxidant activity of the *Trichosanthes* extracts in the livers of diabetic rats was tested using ascorbic acid as control. The results showed that this activity increases with increasing concentration of the extracts. However, in terms of hydroxyl and DDPH radical scavenging, the activity of the water-soluble fraction was greater than that of the ethanol fraction (Figures 4(f) and 4(h)). Meanwhile, in terms of superoxide anion radical scavenging, the activities of the two fractions were comparable (Figure 4(g)).

## 4. Discussion

The experimental results obtained in this study indicate that both the water-soluble and ethanol fractions of *Trichosanthes* can effectively and rapidly reduce the blood glucose levels in diabetic rats. Moreover, these fractions can regulate water consumption and urine excretion in T1DM rats, and long-term intervention also alleviates hyperglycemia symptoms. Although both extracts have a hypoglycemic effect, the underlying mechanisms are different. For instance, the water extract reduces systemic glucose concentration by regulating the glycogen content in the liver of diabetic rats, while the ethanol extract regulates the glycogen in the skeletal muscles. Moreover, the two *Trichosanthes* fractions have comparable antioxidant activities *in vitro*, but the activity of the water-soluble fraction is higher *in vivo*. This indicates that the antioxidant activity of the water extract is the main factor underlying the effect of this fraction in lowering blood glucose, regulating oxidative stress, and relieving diabetes symptoms [20, 21]. Although the liver glycogen content is reduced after ethanol extract intervention, this intervention does not influence the *in vivo* antioxidant activity. Therefore, the hypoglycemic effect of the ethanol extract is controlled by factors other than antioxidant activity.

The main targets of hypoglycemic action are the liver, muscles, lipids, and islet *β* cells [22–24]. The liver is the storehouse of glucose. The liver is one of the organs of insulin. The regulation of blood glucose by the liver depends on the level of blood insulin and its sensitivity to insulin. After eating, blood sugar levels increase, and for those without diabetes, the pancreas increases insulin release into the blood. Insulin sends a signal to cells all over the body to take glucose from the blood. Cells will take glucose as energy and consume it directly, but hepatocytes are different: they store the glucose in the form of glycogen (glycogen synthesis), just like the storage warehouse of excess glucose in the body [25, 26]. Herein, we show that the glycogen content in rat livers increases significantly after the administration of the water-soluble fraction but does not change appreciably upon the administration of the ethanol fraction. Moreover, although the intervention with water-soluble fraction alleviates skeletal muscle atrophy, the effect of the ethanol fraction is significantly better. These results indicate that while the water extract acts on the liver, the ethanol extract acts on muscles. Considering that both extracts can effectively regulate fat and increase insulin secretion, their mechanisms of action must have something in common.

## 5. Conclusion

This study shows that the water-soluble and ethanol extracts of *Trichosanthes* can both reduce the level of blood glucose in diabetic rats; however, the mechanisms underlying the hypoglycemic effects of these two extracts are not the same. This study did not explore the mechanism of the two extracts on inhibiting T1DM. But in later experiments, we will explore the mechanism of two extracts how to control the blood glucose changes in T1DM and find the targets of two extracts on inhibiting T1DM by metabonomics. In fact, we have started this work, and we will continue to show our research results in the future.

## Figures and Tables

**Table 1:** Effects of *Trichosanthes* extract on body weight, fasting blood glucose, drinking water, and urine volume of diabetic rats.

Groups	Body weight (g)	FBG (mmol/L)	Drinking water (mL)	Urine volume (mL)
Sham	388.13 ± 11.19	5.63 ± 0.66	37.51 ± 3.76	14.73 ± 1.52
Model	199.20 ± 5.26^##^	30.2 ± 1.90^##^	190.04 ± 9.76^##^	133.50 ± 17.01^##^
Water-soluble fraction	247.57 ± 20.20^∗∗^	15.43 ± 1.81^∗∗^	102.18 ± 17.81^∗∗^	66.33 ± 15.09^∗∗^
Organic fraction	239.86 ± 21.31^∗∗^	17.25 ± 2.35^∗∗^	104.34 ± 16.35^∗∗^	67.64 ± 10.69^∗∗^

^##^ *p* <0.05 vs. the sham group; ^∗∗^*p* < 0.05 vs. the model group.

**Table 2:** Effects of *Trichosanthes* extract on OGTT.

Groups	0 min	30 min	60 min	120 min	150 min	180 min
Sham	5.88 ± 0.94	7.63 ± 0.86	9.69 ± 1.76	6.98 ± 0.55	6.28 ± 0.59	5.68 ± 0.65
Model	27.62 ± 6.14^##^	31.11 ± 4.14^##^	33.43 ± 8.89^##^	26.28 ± 11.31^##^	24.28 ± 9.52^##^	22.28 ± 10.01^##^
Water-soluble fraction	15.66 ± 4.25^∗∗^	20.43 ± 2.01^∗∗^	25.88 ± 9.33^∗∗^	16.09 ± 6.13^∗∗^	15.26 ± 7.36^∗∗^	15.01 ± 5.66^∗∗^
Organic fraction	16.93 ± 6.09^∗∗^	23.25 ± 2.35^∗∗^	26.34 ± 7.49^∗∗^	18.04 ± 3.85^∗∗^	17.22 ± 4.44^∗∗^	16.14 ± 5.70^∗∗^

^##^ *p* <0.05 vs. the sham group; ^∗∗^*p* <0.05 vs. the model group.

## Data Availability

The data supporting the results of this study has been provided in the manuscript.
